# “Art, Colors, and Emotions” Treatment (ACE-t): A Pilot Study on the Efficacy of an Art-Based Intervention for People With Alzheimer’s Disease

**DOI:** 10.3389/fpsyg.2020.01467

**Published:** 2020-07-16

**Authors:** Federica Savazzi, Sara Isernia, Elisabetta Farina, Raffaella Fioravanti, Alessandra D’Amico, Francesca Lea Saibene, Marco Rabuffetti, Gabriella Gilli, Margherita Alberoni, Raffaello Nemni, Francesca Baglio

**Affiliations:** ^1^IRCCS Fondazione don Carlo Gnocchi ONLUS, Milan, Italy; ^2^Research Unit on Psychology of the Art, Università Cattolica del Sacro Cuore, Milan, Italy; ^3^Università degli Studi di Milano, Milan, Italy

**Keywords:** non-pharmacological intervention, neurodegeneration, visual art, adherence, acceptability, cognition, Alzheimer’s disease, rehabilitation

## Abstract

Increasing evidence suggests that non-pharmacological therapies impact on neuropsychiatric symptoms and quality of life in people with Alzheimer’s disease. Among these, art-based interventions seem particularly suitable for elders’ rehabilitation as they act both on cognitive functions and quality of life. However, their benefits are not yet appropriately explored. The main aim of this quasi-experimental study was to test the feasibility and the likely efficacy of a novel multi-dimensional visual art intervention for people with Alzheimer’s disease (PWAD), named Art, Colors, and Emotions treatment (ACE-t). A group of PWAD (*N* = 10) was recruited from the Memory Clinic of Don Gnocchi Foundation to take part in the ACE-t. A historical control group that followed a usual care program (*N* = 10) was used for comparison. We considered both feasibility output (adherence and acceptability) and efficacy outcome measures (neuropsychological and neurobehavioral scales). We observed a good adherence to and acceptability of the ACE-t. The following significant intervention-related changes were also observed in ACE-t with respect to usual care: improvement in general cognition, as assessed with the Alzheimer’s Disease Assessment Scale–Cognitive, amelioration in language, and in executive functions, and reduction in Neuropsychiatric Inventory Scale score. In conclusion, ACE-t could be considered as a suitable intervention for the rehabilitation of PWAD, with positive effects on the cognitive and the behavioral status. ACE is a promising new art-based intervention that merits further research, including confirmatory trials of our preliminary results.

## Introduction

Alzheimer’s disease (AD) is a complex pathology consisting of a manifold of contributing factors to its etiology (genetic and environmental variables, physical status, psychological well-being, *etc*.) and implying a cognitive decline on multiple structural and functional layers. To date, no effective pharmacological treatments exist to counteract the disease. Thus, research on non-pharmacological interventions with the goal of counteracting the disease and improving the functioning and quality of life is urgent (Livingston and Frankish, 2015). In the recent years, efforts have been made to investigate the beneficial effect of art-based interventions, which include visual art, dance, drama, and music, which demonstrate a positive influence on the functioning of older people and those with cognitive decline (Chancellor et al., 2014; Clark et al., 2018; Windle et al., 2018b; Zhao et al., 2018; Partridge, 2019; Keating et al., 2020). In 2006, the Museum of Modern Art (MoMA) of New York launched the “Meet Me at MoMA,” an innovative art education program purposely for people in the early and middle stages of Alzheimer’s disease. In later years, this project was expanded worldwide at many museums with initiatives for people with AD (PWAD) and their caregivers (CGs) (Rosenberg, 2009). In general, art interventions are based on the so-called aging paradox (Prakash et al., 2014), according to which the progressive atrophy of prefrontal regions, with consequent decline of cognitive processing, is associated with the enhancement of the preserved emotional ones (Bucks and Radford, 2004; Klein-Koerkamp et al., 2012). Thus, the channel of emotion processing becomes a preferential way to access the elders’ interest and facilitate the compensation of other abilities or scaffold the decaying cognitive ones. Studies on esthetics revealed that the observation of visual art elicits in parallel the cognitive interplay between bottom-up and top-down processes as well as emotional and empathetic processing, leading the observer to an emotional reaction and an esthetic judgment (Leder et al., 2004; Massaro et al., 2012; Leder and Nadal, 2014; Savazzi et al., 2014a, b; Gernot et al., 2018). Art appreciation and production are maintained during healthy aging (Kim, 2013; Istvandity, 2017). There is also evidence that the brain mechanisms and the cognitive processes involved in creative art activities are not irreparably damaged in AD (Ehresman, 2013).

In addition, several studies revealed that these activities are suitable also for people with a neurodegenerative decline that affects their functioning at different levels: cognitive, behavioral, and emotional–communicative levels (Chancellor et al., 2014; Czamanski-Cohen et al., 2014; Dunphy et al., 2019; Shafir et al., 2020). At the cognitive level, visual art enhances positive memories by reducing worries about death and ruminative thoughts (Dunphy et al., 2019) and reinforces sustained attention and intellectual engagement (Camic et al., 2014) as well as episodic memories and learning (Eekelaar et al., 2012) in subjects with dementia. In addition, art therapy for older adults has been reported to improve concentration (Kongkasuwan et al., 2016). The dedicated activities stimulating cognition include recalling memories *via* engagement of collage making (Choi and Jeon, 2013) and coloring pre-drawn lines (Peisah et al., 2011). At the behavioral level, art interventions reduce anxiety, agitation, and depression in dementia (Stewart, 2004; Safar and Press, 2011). Also, a case study reports fewer behavioral disturbances and reduced psychiatric medication after an art therapy program (Mimica and Kalinić, 2011). Related activities, such as relaxation and guided imagery (Ciasca et al., 2018), group discussion on emotional expression (Ali et al., 2014), the enhancement of self-expression through different themes (Hsu et al., 2017), and mandala drawing (Kang et al., 2010), are described in art therapy trials. Finally, at the emotional/communicative level, several lines of evidence support the positive influence of art stimulation on emotion management, socialization, and communication (Kang et al., 2010; Flatt et al., 2015; Young et al., 2015). The group setting of art therapy activities provides the participants with a safe environment to share one’s own thoughts and inner experience without necessarily relying on conventional channels of communication. The activities usually include group engagement, development of significant relationships among group members, and self-integration (Kim et al., 2016).

As documented by a recent literature review (Windle et al., 2018a), when multiple areas were stimulated, significant improvements were not found in terms of quantitative measures (Camic et al., 2014). However, the majority of the studies reported positive findings with the use of qualitative outcomes (*e*.*g*.,Camic et al., 2014, 2015; Flatt et al., 2015; Hazzan et al., 2016; Burnside et al., 2017). Moreover, given the paucity of studies investigating the mechanisms of change of art-based intervention on multiple domains in AD, new protocol evaluations with validated measures and quantitative outcomes are needed (de Medeiros and Basting, 2013; Zeilig et al., 2014; Burnside et al., 2017). In fact, even though studies on art perception clearly showed that visual art stimulates bottom-up and top-down cognitive processes (Leder et al., 2004; Massaro et al., 2012; Savazzi et al., 2014b), the efficacy of art treatments on cognitive processes in PWAD is still inconsistent (Chancellor et al., 2014; Young et al., 2016; Deshmukh et al., 2018; Windle et al., 2018a).

Our group has a long and validated experience in the non-pharmacological treatment of people with cognitive impairment and their CGs (Farina et al., 2006a, b; Baglio et al., 2015). Given the above premises on the benefits of art interventions on dementia, we developed a new visual-art-based therapeutic approach termed “Art, Colors, and Emotions” (ACE treatment). In accordance with recent guidelines on the introduction of new rehabilitation interventions (Gitlin, 2013), we designed the ACE-t (ACE treatment) protocol and we preliminarily tested its feasibility and its efficacy in a quasi-experimental study. In this regard, the present study aimed at answering the following research questions: (1) Does ACE-t promote participation (adherence)? (2) Is ACE-t acceptable for its users (acceptability)? (3) Compared to a control group with no ACE-t, does ACE-t efficiently improve the patients’ quality of life, cognition, as well as behavioral symptoms (efficacy)? Findings from the study will allow us to advance a working intervention protocol and to better define art-treatment delivery characteristics to proceed with the evaluation of protocol efficacy against a protocol with a randomized controlled trial (RCT) study and to continue in the analysis of model sustainability.

## Materials and Methods

### Study Design

This study represents the first step to evaluate the potential efficacy of an art-based intervention, ACE-t, on the cognition, behavior, and quality of life of PWAD. A quasi-experimental design with a control group and pretest was adopted to pilot test whether the intervention affects outcomes with a small sample size (Harris et al., 2004; World Health Organization, 2016). Results of this trial will be decisive for the possibility to support the efficacy of the ACE-t to warrant further confirmatory trials, such as RCTs. Two groups were involved in the study: the ACE-t group (experimental group) and the ACE-nt group (control group). Outcome measures were evaluated at baseline and after the treatment in both groups.

### ACE Treatment Description

#### ACE-t Background

The ACE-t treatment implementation was grounded on (1) the recent literature supporting the beneficial potential of arts on different health-related conditions (Czamanski-Cohen and Weihs, 2016; Dunphy et al., 2019), (2) previous experience on rehabilitation (Farina et al., 2006a, b; Baglio et al., 2015) gained by our group, and (3) a preliminary investigation on the opinions of receivers about art-based treatments (see Supplementary Material for additional details).

#### ACE-t Delivery

At an operational level, the intervention was administered by a psychologist with experience in art treatment and a rehabilitation therapist (with the Italian university degree “terapista della riabilitazione”) who specialized in cognitive rehabilitation and trained in our clinic. The enrolled subjects were assigned to mixed-sex groups with six to eight subjects and given 2 h of group sessions twice a week for 7 weeks in a dedicated room at our Memory Clinic. Supervised training in small groups was intended to support effective exercise and to promote social interactions as suggested in the literature (Gates and Sachdev, 2014). In fact, group cohesion was also taken into consideration by inviting the participants to take part in group activities (*e*.*g*., sharing and describing their own photo to the group). Evidence in the literature supports the importance of social stimulation activities as protective factors against major cognitive decline (Karp et al., 2006; Yaffe et al., 2009). The CGs were given two information meetings. The last session with PWAD was open to CGs for the exposition of the works realized during the treatment and for a farewell party.

#### ACE-t Program and Activities

The treatment was structured in 14 sessions aimed at affecting patients on different areas (cognition, behavior, and communication), in line with a holistic approach (Chancellor et al., 2014; Young et al., 2016; Windle et al., 2018a).

The use of primary colors in paintings by artists of the XIX and XX centuries was the guiding theme of the program. The choice was motivated by the following considerations: (1) art of the nineteenth and twentieth century abandons representative style in favor of intense color and simplified shapes to express the inner life of the artist and (2) color conveys many cultural and personal emotions and meanings. The use of color is an immediate, non-verbal way to express inner thoughts, feelings, and emotions when words are lacking.

The program was intended to lead participants from the acquisition of knowledge regarding the meaning of primary colors and their possible combinations to the use of color to express memories and internal experiences. We therefore started from an explanation of the primary colors and their possible combinations as well as the meanings and emotions that they convey through the assistance of a painter and his work. The program aimed at leading the participants to the autonomous production of their canvas and the exhibition of artworks to the other members of the group and their caregivers.

We worked on different domains (cognition, behavioral symptoms, and communicative abilities) to actively reduce potentially negative stress factors. An alternation of six phases with specific activities was conceived for each of the 14 ACE-t sessions. In particular, (1) activities to promote cognition were meant to reinforce specific cognitive sub-domains: language, procedural, semantic, and autobiographical memory, executive functions, and attention; (2) activities to reduce behavioral symptoms were meant to offer a rich multisensory stimulation to reduce agitation and apathy and reinforcing alternative strategies for the recognition of objects and the recall of procedures; and (3) activities to enhance communication aimed at promoting the expression of internal emotions through verbal and non-verbal languages and the recognition and expression of others’ and self’s emotions.

More specifically, the activities in each of the 14 sessions were always sequentially framed in six phases:

(1) First is the “welcoming” phase (lasting about 10 min), in which visual supportive material helped the participants to recall the activities of the previous session. Then, the color theme that would guide the meeting (“color theme of the day”) was presented with the use of sensorial stimuli (*e*.*g*., yellow fruits and vegetables, autumnal leaves, red flowers, powder spices, blue paper cuts, *etc*.).
(2) In the “color and materials” phase (20 min), the participants were encouraged to make a sensory experience of objects and materials, to name them, and to explain the difference between several hues of the color. They were also asked to make a link between the object/material and the way they used it in everyday life or to collect and share memories elicited by it (*e*.*g*., cooking recipes, a well-known autumn landscape, and how to water garden plants).
(3) In the “meeting with the artist” phase (about 30 min), the biography of a painter was presented, starting from the analysis of one of her/his paintings. The artwork was always chosen in relation to the color and the materials previously analyzed. The lives of painters and the characteristics of the painting under analysis were a starting point for reflecting together on events and emotions that may be close to the participants’ own experiences.
(4) Next, a brief “occupational break” (about 10 min) was planned. When freely agreeing, the participants actively contributed in preparing the atelier materials for the next phase.
(5) The “practical activity” phase (40 min) was conceived to let the participants exercise in the acquisition of simple painting techniques (*e*.*g*., to mix tempera colors, to fill in the chromatic circle, and to cut and paste cut paper) or to help the participants connect to their emotions and memories by means of acquired pictorial skills (painting or drawing landscapes and objects relevant for one’s own experience). Soft background music was played to create a comfortable environment and to foster engagement with the task.
(6) The remaining 10 min was the “conclusion” phase, in which the participants shared their work and emotions with others and collaborated in tidying up the room.

In Table 1, the sequencing of the activities, sessions, and weeks is detailed, and in Table 2, examples of activities in relation to the specific domain for which they were conceived are presented. In Table 2, the specific phase of the session in which each kind of activity was delivered is also illustrated.

**TABLE 1:** Art, colors, and emotions activities per week, session, and session phases.

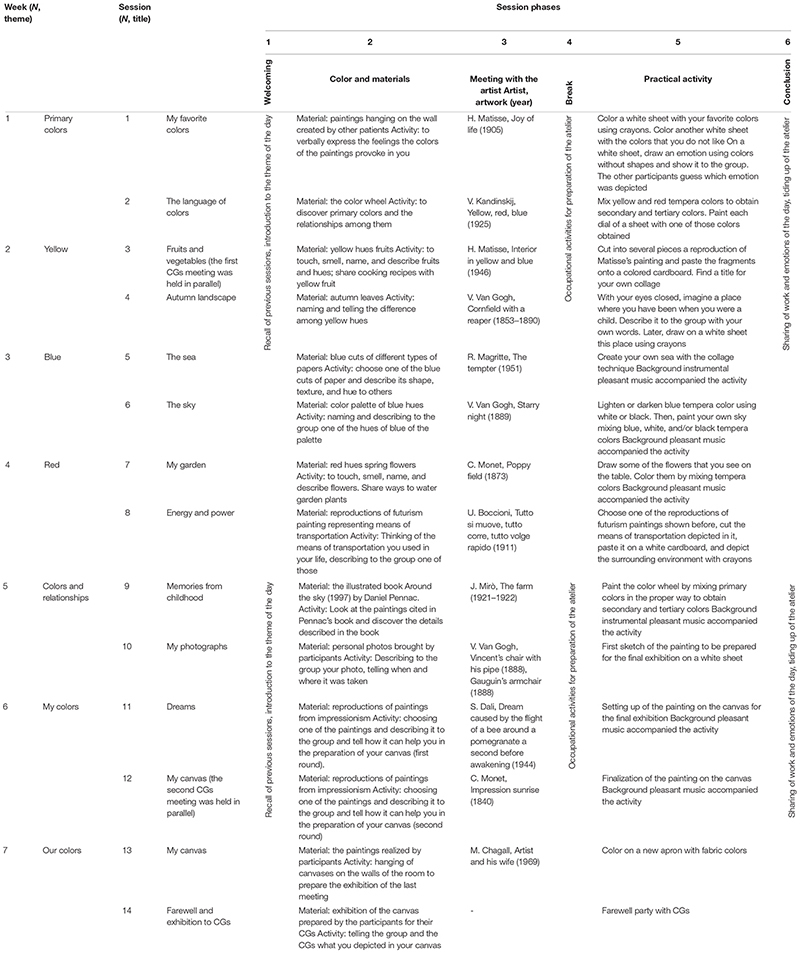

*CG, caregiver, N = number.*

**TABLE 2:** Art, Colors, and Emotions treatment main domains and activities: examples of contents.

Areas	Sub-domain	Example of activity	Session phases					
			1	2	3	4	5	6
Activities to improve cognition	Language	Naming objects, materials, and shapes	X	X	X			
		Naming primary, secondary, and complementary colors	X		X		X	
		Naming emotions			X			X
		Comprehension of simple instructions	X	X		X	X	
	Memory	Recall of popular paintings	X		X			
		Recall of the activities of the week	X					
		Use of the chromatic circle for the memorization of primary colors		X			X	
		Use of the chromatic circle for the recall of the making of secondary colors		X			X	
		Carefully look at a painting and memorizing			X			
		Recall of information from a painting previously observed	X		X			X
		Recall of events, people, and environments from the past		X			X	
	Executive functions and praxis	Put parts of a painting in the correct position					X	
		Mix tempera colors to lighten or darken colors					X	
		Mix tempera primary colors to obtain secondary and tertiary colors					X	
		Fill an empty chromatic circle by placing colors in the right order					X	
		Design of drawings					X	
		Follow simple instructions to use painting materials					X	
	Attention	Found specific details in a painting			X			X
		Tell the difference between different hues of color		X	X			X
Activities to reduce behavioral symptoms	Sensory stimulation	Touch materials of different textures		X				
		Touch, smell, and taste fruit/spices		X				
		Touch and smell flowers and leaves		X				
		Listen to some music related to the theme of the session			X			
		Listen to some music while drawing or painting					X	
Activities to promote communication	Verbal communication	Describing a place from childhood		X				X
		Describing one’s own photo		X				X
		Recognition and description of the emotions, objects, and subjects depicted by other participants						X
		Recognition and descriptions of the emotions, objects and subjects expressed in paintings			X			X
	Non-verbal communication	Drawing and coloring a place from childhood					X	
		Drawing and coloring my favorite objects					X	
		Coloring an emotion					X	

### Participants

Twenty participants, divided into two groups, were considered for the study. The first group was composed of 10 PWAD recruited at the Memory Clinic of Don Gnocchi Foundation of Milan and consecutively assigned to the ACE-t group. The second group comprised of a historical control group of PWAD (*N* = 10; ACE-nt group) that followed our Memory Clinic’s usual care program (*e*.*g*., recommendations on healthy nutrition, the correct dose of physical activity, and stimulating activities at home). The ACE-nt group was taken from the participants in the usual care program of the study of Baglio et al. (2015). The PWAD of the ACE-t group were matched with participants to the usual care program of the previous study (Baglio et al., 2015) on the basis of socio-demographic characteristics (age, sex, level of education, and Mini Mental State Examination) (Folstein et al., 1975; see Table 3).

**TABLE 3:** Domains addressed by primary and secondary outcome measures in the experimental (ACE-t) and the control (ACE-nt) groups.

Domains	Outcome	Measure
		
Quality of life	Primary	QoL-AD
Behavior	Primary	NPI
Cognition	Primary	ADAS-cog
	Secondary	Verbal fluencies (phonemic and semantic)
	Secondary	Token test
	Secondary	Attentive Matrices

*ADAS-cog, Cognitive Subscale of the Alzheimer’s Disease Assessment Scale; NPI, Neuropsychiatric Inventory; QoL-AD, Quality of Life Alzheimer’s Disease Scale.*

The outpatients’ eligibility criteria were the following: a diagnosis of probable AD according to NINCDS ADRDA revision criteria (McKhann et al., 2011), a mild phase of disease according to the Clinical Dementia Rating scale (range 0.5–1) (Morris, 1997), a minimum of 5 years of school attendance, and age over 65 years old. The exclusion criteria were absence of CG, patients with other types of dementia, major psychiatric disorders, and a serious cognitive decline that would prevent them from properly answering or completing the questionnaires and scales.

All the participants gave their informed written consent to participate in the study. The Ethics Committee of Don Gnocchi Foundation of Milan approved the study.

### Measurement

*Adherence* to ACE-t only was estimated considering the attendance rate to the protocol for each participant, and in case of absence, the justification was registered.

To test *acceptability*, an adaptation of the Greater Cincinnati Chapter Well-Being Observational Tool©(GCWBT) (Kinney and Rentz, 2005; Sauer et al., 2016) was used. Social–behavioral patterns were assessed once a week on a group of PWAD (*n* = 5) during ACE-t only. The tool is composed of seven domains categorized into two major domains: well-being (social interest, engagement, and pleasure) and ill-being (disengagement, negative affect, sadness, and confusion). Two independent trained observers recorded for each domain the frequency of every single indicator on a five-point Likert scale (0 = “never”; 4 = “most of the time”). For analyses, we used the subscales “engagement” and “disengagement”, considering a session at the beginning of the treatment (the 4th session) and a session at the end of the treatment (the 13th session) (inter-rater agreement, weighted Kappa = 0.72, standard error = 0.04). According to Sauer et al.(2016, p. 7) (Berry and Pennebaker, 1993), the engagement scale measures when a “participant is able to attend to project or activity for 5 min at a time; participant stays focused on the task at hand; ideally enters a state of flow or total engagement; engages with others for task-related support and initiates in task-related conversations; participant may engage in conversation with facilitator during the activity but major focus is task-related.” The disengagement scale is focused on measuring when a “participant is not engaged in the activity; stares down or into space; falls into a deep sleep; leaves the activity area.”

To test the *efficacy* of ACE-t, we compared the results to primary and secondary outcome measures between the two groups (ACE-t *vs* ACE-nt). The PWAD underwent a neuropsychological assessment at 1 week before (baseline) and after (T_1) the treatment by a neuropsychologist blinded to the study. The PWAD and CGs were instructed not to discuss the nature of the therapy with the research assistants who did the assessments. Each research question was addressed with specific measures. Because of the multidimensionality of the study, primary outcome measures were assessed through one primary outcome measure for each domain investigated:

(1) The Quality of Life Alzheimer’s Disease scale (QoL-AD) (Logsdon et al., 2002), administered both to patients and CGs, was considered as the primary outcome measure of the quality of life domain.
(2) The Neuropsychiatric Inventory (NPI) (Cummings et al., 1994; Cummings, 1997) consisted of the primary outcome measure of the behavioral domain, assessing the frequency and the impact of behavioral symptoms.
(3) The Cognitive subscale of the Alzheimer’s Disease Assessment Scale (ADAS-cog) (Fioravanti et al., 1994) was the primary outcome measure of the cognition domain. In addition to ADAS-cog, cognition was evaluated also with secondary outcome measures: verbal fluencies (Novelli et al., 1986)—phonemic (F, P, L) and semantic (animals, fruits and cars)—to test lexical access, the Token Test (Spinnler, 1987) to test language comprehension, and Attentive Matrices (Cummings, 1997) to test visual attention.

Table 3 reports the primary and the secondary outcome measures related to each domain addressed.

### Statistical Analyses

Statistical analyses on outcome measures were performed using MedCalc software (Version 14.8.1).

Shapiro–Wilk normality test was run to verify whether data were parametrically or non-parametrically distributed. Accordingly, parametric and non-parametric tests were applied to compare groups’ baseline characteristics as appropriate.

To test the efficiency of ACE-t, the rate of adherence to the treatment was calculated by summing up the number of participants attending each session. Also, indexes of the GCWBT of each participant at each session were calculated. Then, scores of GCWBT were compared between the baseline and the end of the treatment with the Wilcoxon signed-rank test.

To test the efficacy, changes in neuropsychological test and scale scores from baseline (delta changes: T1-baseline) were calculated. Then, a comparison between ACE-t and ACE-nt group was performed through Mann–Whitney non-parametric comparison. Non-parametric pairwise comparisons (Wilcoxon signed-rank test) were also performed in each group. All statistical analyses were two-sided, with an α-value of < 0.05 considered as statistically significant. Considering the preliminary nature of the study, the power of each outcome in relation to all outcome variables was not estimated. The results will define the parameters to calculate the power to perform statistical hypothesis testing in future studies.

## Results

The two groups were similar at baseline in socio-demographic characteristics, behavioral symptoms, and global cognitive level. The baseline characteristics are reported in Table 4.

**TABLE 4:** Participants’ baseline demographics and neuropsychological characteristics.

Demographic characteristics	ACE-t participants	ACE-nt Participants	Group comparison
*N*	10	10	n.s.
Age, years (mean ± SD)	79.10 ± 4.53	77.80 ± 5.51	n.s.
Gender (male:female)	3:7	3:7	n.s.
Level of education, years (mean ± SD)	9.40 ± 2.91	9.00 ± 2.11	n.s.
MMSE (mean ± SD)	22.30 ± 4.11	23.20 ± 1.93	n.s.
NPI frequency (mean ± SD)	9.66 ± 4.32	17.30 ± 11.64	n.s.
NPI distress (mean ± SD)	5.66 ± 2.26	8.70 ± 6.57	n.s.

*ACE-t, people treated with “Art, Colors, and Emotions” treatment; ACE-nt, people with the usual care program; MMSE, Mini-Mental State Examination; N, number; NPI, Neuropsychiatric Inventory Scale; n.s., not significant; sd, standard deviation.*

### ACE-t Acceptability

The preliminary investigation on the opinion of people with cognitive decline and their caregivers highlighted a positive feedback regarding the acceptability and the suitability of the art-based treatments for patients’ residual abilities and in terms of being estimated as useful. Also, these interventions were judged interesting and pleasant by users, supporting their acceptability. The deepened results regarding the opinions of receivers are illustrated in the Supplementary Material (ACE-t background). Augmented well-being and compliance to the treatment were confirmed by results from GCWBT, showing that the participants’ disengagement attitude significantly decreased at the end of the ACE-t (*N* = 5; beginning session: mean = 1.15, SD = 1.04; end session: mean = 0, SD = 0; *p* < 0.001).

### ACE-t Adherence

The participants in the ACE-t group showed good adherence to the treatment, completing the 14 sessions and attending the program with assiduity. The majority of the participants (six out of 10) did not miss a session. For the remaining four, the non-attendance rate was lower than 25% of the sessions (maximum of three sessions missed) because of illness and/or of overlapping appointments. The CGs informally referred to the staff that the participants gladly took part in the intervention.

### ACE-t Efficacy

The results obtained by PWAD and the comparison between the two groups (ACE-t *vs*. ACE-nt) on tests and scales are detailed in Table 5 and Table 6.

**TABLE 5:** Within-group and between-groups comparison of results on pre- and post-treatment primary outcome measures.

	ACE-t participants			ACE-nt participants			Group comparison
Domain	Median (25th–75th percentile)		Wilcoxon	Median (25th–75th percentile)		Wilcoxon	Mann–Whitney *U*
Test, subscales	Pre-treatment	Post-treatment	*p*	Pre-treatment	Post-treatment	*p*	*p*
**General cognition**							
ADAS-COG	28.00 (25.00–36.00)	22.50 (18.00–26.00)	*0.002*	21.80 (17.60–24.00)	21.00 (17.30–26.00)	0.625	**
**Behavioral**							
NPI							
Frequency	10.33 (7.00–11.00)	1.50 (1.00–5.00)	0.084	17.00 (6.00–23.00)	23.50 (14.00–27.00)	0.275	*
Distress	5.83 (4.00–8.00)	1.00 (0.00–2.00)	0.084	7.00 (4.00–12.00)	8.00 (5.00–14.00)	0.547	**
**Quality of life**							
QOL							
PWAD	31.25 (27.00–36.00)	35.33 (31.00–37.00)	*0.027*	32.50 (26.00–36.00)	32.44 (28.00–38.00)	1.00	-
CG	31.00 (29.00–33.00)	31.50 (26.00–37.00)	0.232	30.50 (28.00–34.00)	29.50 (26.00–31.00)	0.734	-

ADAS-Cog, Alzheimer’s Disease Assessment Scale; ACE-t, people treated with “Art, Colors, and Emotions” treatment; ACE-nt, people with the usual care program; NPI, Neuropsychiatric Inventory Scale; QOL, quality of life; PWAD, people with Alzheimer’s disease; CG, caregiver. Statistically significant *p* values (*p* < 0.05) are reported in italics. *p ≤ 0.05; **p ≤ 0.01.

**TABLE 6:** Within-groups and between-groups comparison of results on pre- and post-treatment secondary outcome measures.

	ACE-t participants			ACE-nt participants			Group comparison
Domain	Median (25th–75th percentile)		Wilcoxon	Median (25th–75th percentile)		Wilcoxon	Mann–Whitney *U*
Test, subscales	Pre-treatment	Post-treatment	*p*	Pre-treatment	Post-treatment	*p*	*p*
**Language**							
Token test	25.75 (21.74–29.81)	29.25 (26.5–31.76)	*0.002*	29.50 (28.50–30.50)	29.25 (26.50–30.50)	0.383	**
Phonemic fluencies	22.50 (14.47–28.62)	23.50 (21–36.57)	*0.009*	26.00 (22.00–29.00)	24.5 (20–30)	0.922	-
Semantic fluencies	12.00 (8.47–18.62)	18.00 (12.42–23.52)	*0.006*	23.50 (20.00–25.00)	18 (16–23)	0.301	**
**Executive functions**							
Attentive matrices	35.50 (28.47–49.57)	39.50 (29.27–50.57)	0.232	42.00 (34.00–50.00)	37.5 (28–42)	*0.019*	*

*ACE-t, people treated with “Art, Colors, and Emotions” treatment; ACE-nt, people with the usual care program. Statistically significant *p* values (*p* < 0.05) are reported in italics.*

### Primary Outcome Measures

A significant increase in the ACE-t participants’ quality of life was found. Behavioral symptoms showed a significant improvement in the behavioral domain in ACE-t participants with respect to ACE-nt participants as measured by NPI (frequency, *p* = 0.014; distress, *p* = 0.006). As for cognition, the ACE-t participants had a significant decrease of the total score in the ADAS-cog scale. The ADAS-cog scores were significantly reduced in the ACE-t group with respect to the ACE-nt group (ADAS-cog score *p* < 0.001).

### Secondary Outcome Measures

Significant improvements in phonemic and semantic fluencies and in Token test were found in ACE-t participants. There was also evidence of a significant improvement in Token test scores, semantic fluencies in ACE-t, and attentive matrices in ACE-t participants with respect to ACE-nt (Token test score *p* ≤ 0.001; semantic fluencies score *p* = 0.009; attentive matrices score *p* = 0.021). The ACE-nt subjects significantly deteriorated in terms of executive functions.

## Discussion

A growing body of scientific evidence sheds light on the potential value of art media integration in health interventions for PWAD (Chancellor et al., 2014; Clark et al., 2018; Windle et al., 2018b; Zhao et al., 2018; Partridge, 2019; Keating et al., 2020). Pivotal studies in the field of art therapy highlight the beneficial effects of art-based interventions on multiple domains of PWAD’s well-being, such as cognition, functioning, and psychosocial abilities (Chancellor et al., 2014). Given the residual creative art-making abilities of PWAD (Ehresman, 2013), working on the channel of emotions through art is particularly recommended as a non-pharmacological intervention, facilitating the maintenance of residual cognitive abilities, slowing down psychiatric symptoms, and enhancing the quality of life. However, the evaluation of art-based interventions for PWAD has produced sparse and non-generalizable evidence until now. Therefore, the present study consists of a proof-of-concept of the efficacy of ACE-t, a new art intervention protocol to enhance the quality of life, behavior, and cognition in elderly people with AD.

First, ACE-t was evaluated by testing its adherence and acceptability. Our study documented a high adherence to ACE-t treatment both for PWAD and their CGs. This study also yielded an enhancement of PWAD’s well-being by reporting the participants’ decreased disengagement at the end of the treatment. This result is in line with most studies on the outcomes of art treatments that have reported positive effects on this domain (Chancellor et al., 2014; Zeilig et al., 2014; Young et al., 2016; Windle et al., 2018b). Along with the sessions, the ACE-t participants progressively acquired awareness of being part of a group, and they exercised basic graphic competences that allowed them to feel engaged in group activities. Considering the large number of barriers, such as physical limitations, low energy, and burden of the caregivers and the facilitators, including the perceived benefits and socialization on non-pharmacological interventions for people with dementia (van Alphen et al., 2016; van der Wardt et al., 2017), we may declare that ACE-t displays prerequisites for facilitating increased adherence and motivation for PWAD. It is well known that motivation is an extremely important factor to reach the desired outcome. In line with this, our data support the feasibility of this kind of intervention with PWAD.

Regarding efficacy results, this preliminary study supported the hypothesized efficacy of ACE-t on multiple AD domains: quality of life, reduction of behavioral symptoms of dementia, and positive effects on cognitive functions. Considering the quality of life, we observed an improvement from the baseline into the group of participants to ACE-t, although a between-groups effect was not observed.

The participants reported a significantly higher quality of life as measured by D-QoL scales after the treatment. This finding is in line with a study by Hattori et al. (2011) that reported vitality and quality of life benefits from frequent sessions of art activities against studies with only weekly 1-h sessions. This suggests that an intensive rehabilitation approach, such as ACE-t, should have a higher efficacy on the quality of life dimension than the one with fewer weekly sessions. As underlined by a recent review on art-based intervention in older adults, longer-term approaches, including at least 12 weeks of treatment, are able to stimulate relationship building and aid in sustaining the outcome achievements (Dunphy et al., 2019). Improvement in the quality of life could be also related to the peculiarity of art interventions of being based on visual materials. When words are lacking, the non-verbal language of visual art offers preferential access to one’s own inner world and to the expression of feelings. Verbal and non-verbal expressions of emotions are associated with reductions in autonomic nervous system activity and bear some relation to well-being and health (Spector et al., 2011). The positive effect of non-verbal expressive techniques is most important to preserve well-being when language ability is decaying. The mechanisms of change implied in visual art intervention especially involve the processing of emotion by stimulating the externalization of an implicit sensation *via* pictorial representation (Czamanski-Cohen and Weihs, 2016), without necessarily relying on verbal cognitive skills. The engagement with art material and the stimulation of visual imagery lead to a preferential access to the emotion channel than a verbal one, drawing a way to intervene to compensate or scaffold the cognitive abilities of PWAD (Prakash et al., 2014).

As far as the behavioral domain is concerned, the reduction of symptoms with ACE-t is not surprising. Indeed the most common outcome from non-pharmacologic approaches in the treatment of dementia is an improvement in the management of the behavioral and the psychological symptoms in dementia. It was observed in previous studies on multidimensional therapy carried out by our group (Farina et al., 2006a, b; Baglio et al., 2015). It was also accounted for by other non-pharmacological art treatment studies (Berry and Pennebaker, 1993; Stewart, 2004; Rusted et al., 2006; Teri et al., 2008; Hattori et al., 2011; Spector et al., 2011; Henry et al., 2016; Zucchella et al., 2018), reporting a reduction of agitation, depression, and apathy as well as improved affect in people with dementia. Particularly, physical relaxation and stress reduction are the mechanisms of change related to visual art interventions reported in the literature (Hsu et al., 2017; Dunphy et al., 2019). Art interventions are especially demonstrated to have a considerable efficacy on reduced depression experience in older adults (Im and Lee, 2014; Dunphy et al., 2019). The marginal inclusion of music listening during art-making activities of ACE-t could have also provided beneficial effects by slowing down the negative affect, as is documented in healthy older and younger adults (Groarke and Hogan, 2019). Moreover, literature highlights that creativity in dementia contributes to help to copy symptoms related to the disease as well as express emotions and the inner self (Palmiero et al., 2012). The non-pharmacological management of the behavioral and psychological symptoms of dementia (BPSD) is clinically significant because it may help to reduce the use of antipsychotic drugs with PWAD (Ballard et al., 2009). Furthermore, the non-pharmacological management of BPSD is recommended and cost-effective as it can have positive effects on the quality of life and the satisfaction of the patient–caregiver dyad (Gitlin et al., 2010).

Regarding cognition, we found an improvement in general cognitive functions, in executive functioning and in language access, and comprehension. These results seem to be innovative in relation to studies assessing cognition in art treatment *versus* the control groups (Berry and Pennebaker, 1993; Rusted et al., 2006; Hattori et al., 2011) where no changes in the cognitive domains were found with art therapy. Several mechanisms of change in cognition related to visual art interventions have been reported in the literature (Dunphy et al., 2019), including strengthening positive memories and reducing ruminative thoughts. Evidence from qualitative studies suggests that visual art intervention contributes to enhance learning and memory abilities (Camic et al., 2014). It is interesting to notice the specific influence of ACE-t on language, also in comparison with usual care treatment, since art therapy leverages mostly on non-verbal expression. Nevertheless, ACE-t was a group-based intervention and included activities aimed at reinforcing communication and the expression of memories and emotions. The benefits from participating in a group for PWAD are related to social interaction and the creation of significant relationship networks, consequently promoting language function (Spector et al., 2011) and the perception of overtaking isolation (Teri et al., 2008). In this sense, ACE-t allowed the participants to share and frame their emotions and to discuss their esthetic appreciation *via* not only non-verbal but also verbal channels in a group setting. The prevention of worsening language abilities is a clinically important result as it favors the interaction with CGs, encouraging them to bear the burden of care. Furthermore, considering that successful communication is based on social cognitive skills, ACE-t may also positively affect socio-cognitive function. In future studies, it will be interesting to test the ACE-t efficacy also on this relevant domain for mental health and well-being (Henry et al., 2016).

Interestingly, the effect of the treatment was sustained by the spontaneous creation of a mutual support network by CGs, such as organizing cars to bring a group of PWAD to the center for the ACE sessions or planning common initiatives outside of treatment. In this regard, the enhancement of socialization in art intervention is well demonstrated. In fact, the setting of the treatment consists of small group work, intrinsically encouraging socialization and sharing (Kim et al., 2016; Kongkasuwan et al., 2016; Dunphy et al., 2019).

Overall, despite the significant results in improving the different aspects of PWAD functioning, our study is not without limitations. First of all, our sample receiving ACE-t was compared with a prior historical control sample following a usual care program, possibly affecting internal validity. Nevertheless, the new and old samples were comparable and well-characterized at baseline. Furthermore, since the cost of testing the efficacy of interventions is high, our study was conceived as a first step in the definition of the parameters to calculate the effect size with which to perform an RCT study. Future studies should examine ACE-t efficacy on multiple domains over time and with a larger cohort of participants at different stages of disease development, considering also people in the preclinical phases of neurodegeneration, such as in mild cognitive impairment. In fact, the small sample size could have contributed to not reaching significance in the between-groups effect in quality of life measure, although a significant increment was registered in the ACE-t group. Another limitation of the current study also consists of not considering the art background as a criterion for the participant’s enrolment. Finally, considering the GCWBT inter-rater agreement, according to recent interpretations (McHugh, 2012), a Cohen’s Kappa between.60 and.79 is considered as moderate, not substantial, and thus ameliorable. Considering these limitations, our results encourage future RCTs, taking into account also the art background of subjects as a criterion of enrolment and including structured qualitative evaluations to collect the impressions and the suggestions of the participants. These data will drive the next steps to modify the intervention in the future.

The added value of this kind of non-pharmacological intervention is that it is not limited to mere cognitive training but open also to the promotion of well-being in an enriched relational environment with significant others. The active involvement of CGs, group participation promotion, activities focused on residual abilities rather than on deficiencies, emotion-evocative visual art stimuli, and engaging activities altogether had success in reviving and reinforcing PWAD’s relational context, also affecting multiple functioning dimensions.

To conclude, ACE-t represents a feasible and acceptable non-pharmacological intervention for PWAD and deserves further research for its validation.

## Data Availability Statement

The datasets generated for this study are available on request to the corresponding author.

## Ethics Statement

The studies involving human participants were reviewed and approved by local Ethics Committee of Don Gnocchi Foundation of Milan. The participants provided their written informed consent to participate in this study.

## Author Contributions

FB, FS, and SI conceived the study. EF, MA, and RN performed the clinical enrolment and the clinical assessment. AD’A, FLS, and MR contributed to the neuropsychological assessment. FS, GG, and RF performed the rehabilitation activities of the study. FB, FS, and SI performed the statistical analysis. FB, FS, RN, and SI performed the results discussion and the data interpretation. FS and SI wrote the manuscript. All the co-authors reviewed and approved the final version of the manuscript. All authors contributed to the article and approved the submitted version.

## Conflict of Interest

The authors declare that the research was conducted in the absence of any commercial or financial relationships that could be construed as a potential conflict of interest.
