# Supplementary material for: “Art, Colors, and Emotions” Treatment (ACE-t): A Pilot Study on the Efficacy of an Art-Based Intervention for People With Alzheimer’s Disease
Source: Front Psychol. 2020 Jul 16;11:1467. doi: 10.3389/fpsyg.2020.01467 (PMC7378782; doi:10.3389/fpsyg.2020.01467)
Supplement: Supplementary file 1 [file Data_Sheet_1.docx]

Supplementary Materials

**“Art, Colors, and Emotions” treatment (ACE-t): pilot study on the efficacy of an art based intervention for people with Alzheimer’s disease**

**Suppl 1: ACE-t background - Acceptability of art interventions for people with cognitive decline.**

# Introduction

# This study aimed to preliminarily investigate the opinion of people with cognitive impairment and their caregivers on an art intervention suitability and acceptability considering interests and residual abilities of receivers. We followed Orsmond and Cohn (2015) guiding questions to investigate the acceptability and suitability of an intervention and study procedures. With this focus, *ad-hoc* questionnaires were administered to outpatients with cognitive impairment and their caregivers from our Memory Clinic (IRCCS Don Gnocchi Foundation, Milan).

# Method

## Participants

## A sample of 80 participants - composed of people with cognitive impairment (Care Recipients, CRs; n = 40) and their caregivers (CGs, n = 40) - afferent to the Don Gnocchi Foundation Memory Clinic was recruited. CRs were consecutive outpatients with MCI (Mild Cognitive Impairment) according to Petersen (2004) (Mini-Mental State Examination, MMSE (Measso et al., 1993) - score equal or higher than 24) or with AD in mild to moderate phases (mild AD, MMSE score between 21 and 23; moderate AD, MMSE score between 11 and 20) according to McKhann et al. (McKhann et al., 2011), a minimum of 5 years of school attendance, and age over 60 years old. Exclusion criteria were: the absence of CG, major psychiatric disorders, and a severe cognitive impairment that would not enable CR to answer to questionnaires and scales correctly. All participants gave their informed written consent to participate in the study. The local Ethics Committee of the Don Carlo Gnocchi Foundation approved the study.

## Measurement

## All subjects answered to an ad-hoc questionnaire on the CRs’ attitudes to art treatments. The questionnaire was designed to collect CRs’ and their CGs’ opinions on the acceptability and suitability of a visual art-based intervention for the treatment of people with cognitive impairment. Two versions of the questionnaire were prepared, one for CGs (Version A) and another simplified for CRs (Version B). The questionnaire is detailed below (see also table 2). It was developed following the suggestion by Orsmond and Cohn [15] to evaluate the acceptability and suitability of treatment in its initial phases.

## *Version A - Attitude to art treatments questionnaire for CGs*

## The intervention acceptability was investigated utilizing the following variables:

## · CRs’ access to the art entertainments of major success in Italy (going to the museum, going to art expositions, going to the theatre, cinema, ballets, concerts, listening to some music, sewing, drawing, painting, playing an instrument, dancing) [range 0-12];

## · Judgments on (1) perceived utility of an art intervention for people with cognitive impairment, (2) expected benefits from this kind of intervention for their CR, (3) level of interest of their CR in this kind of intervention. For each one of the three judgments, answers were registered on a 9-point-Likert’s scale (1 = not at all, 9 = very much) and later categorized for score level (1-3 = low; 4-6 = medium; 7-9 = high);

## · Justification of the interest of the CR’s towards an art intervention with an open-ended answer. Two independent judges blinded to the study clustered the justifications into four thematic categories (inter-rater reliability K = .87): 1) art is/is not stimulating for the CR; 2) the CR is/is not interested in the arts because of her/his disposition; 3) the CR is/is not interested in an art treatment because of past similar experiences; 4) the CR can/ cannot take part to an art treatment at out Clinic because of the distance from their home. The doubtful cases were collegially resolved.

## The suitability of a visual-art intervention with persons with cognitive impairment was also assessed by asking CGs to rank on a 5-point-Likert’s scale (0 = I don’t know; 1 = not at all; 2 = little; 3 = a lot; 4 = very much) the CRs’ residual abilities in doing eight kind of activities typical of a group art intervention. For analyses, the eight activities were included in three main categories: 1) motor skills - range 0-12 (grasp, release and throw objects; draw and color with different tools; glue and cut); 2) perceptual skills - range 0-8 (handle objects to obtain sensorial information; recognize size, shape, and color of an object); 3) participation skills - range 0-12 (take part to group discussions and activities when encouraged, follow simple instructions; pay attention to or bear an activity of interest for 30 minutes).

## *Version B - Attitude to art treatments questionnaire for CRs*

## The simplified version of the questionnaire also consisted of two parts assessing the intervention acceptability and suitability. As for the intervention acceptability, it was investigated utilizing the following variables:

## · CRs’ access to the art entertainments of major success in Italy (going to the museum, going to art expositions, going to the theatre, cinema, ballets, concerts, listening to some music, sewing, drawing, painting, playing an instrument, dancing) [range 0-12];

## · Judgments on their attitude towards an art treatment ranked on a 9-point-Likert’s scale.

## As for the suitability of an art treatment, CRs were asked to express whether they were compliant or not with eight typical activities of the art treatment. For analyses, answers were included in three categories: 1) Interest in art production activities (drawing with different tools; coloring with various tools; gluing; cutting with scissors); 2) Interest in group activities (taking part to a discussion; taking part to group activities); 3) Enjoying art (listening to music; attending to art meetings).

## Statistical analyses

## To measure the internal consistency of questionnaires subscales Cronbach’s alpha was used. To account for variable normality the D’Agostino-Pearson’s test was performed; parametric or nonparametric analyses were carried out as appropriate. Continuous variables were described by mean and standard deviation values. Categorical variables were described by percentages and multinomial proportions were applied. The Spearman’s rank correlation coefficient was used to assess the degree of accordance in the dyads and the level of correlation between variables related to the access to art entertainments. All statistical analyses were performed with MedCalc software (Version 14.8.1).

# Results

To perform analyses, we excluded five dyads because one of the two questionnaires of the dyads was lacking (questionnaire A = 3; questionnaire B = 2). The characteristics of the included subjects are reported in table 1.

|  | *Dyads* | |
| --- | --- | --- |
|  | *CRs* | *CGs* |
| Subjects (n) | 35 | 35 |
| Age y, mean (sd) | 77(7.56) | 62(12.03) |
| Male:Female (n) | 14:21 | 13:22 |
| Education y, mean (sd) | 8.14(3.55) |  |
| MMSE score, mean (sd) | 23.52(3.63) |  |
| MCI (N = 19) | 25.00(1.24) |  |
| Mild AD (N = 8) | 22.00(0.93) |  |
| Moderate AD (N = 8) | 16.88(2.70) |  |
| Daily time spent with the CR h, mean (sd) |  | 13.07(10.25) |
| Degree of kinship with the CR |  |  |
| Son/daughter and nephew, n (%) |  | 13(37.14) |
| Siblings, n (%) |  | 2 (5.71) |
| Spouse, n (%) |  | 17(48.57) |
| Relatives in law, n (%) |  | 2(5.71) |
| Care-taker, n (%) |  | 1(2.86) |

**Table 1.** Characteristics of participants. *Abbreviations:* AD, people with Alzheimer’s Disease; CG, Caregiver; CR, Care Recipient; MCI, Mild Cognitive Impairment; MMSE, Mini Mental Examination Test; n, Number; sd, Standard Deviation.

A good internal validity of the questionnaire subscales was attested by alpha-levels comprised between 0.7 and 0.9. The results are shown in Table 2.

| *Questionnaire version* | *Domain* | *Scale* | | *α* |
| --- | --- | --- | --- | --- |
| CG | **Intervention acceptability** | **CRs’ access to art entertainments** | | n.a. |
|  |  | **Art treatment judgments** | | 0.91 |
|  |  |  | Perceived utility of art treatment for people with cognitive impairment |  |
|  |  |  | Expected benefit of art treatment for CR |  |
|  |  |  | Interest of CR in art treatment |  |
|  | **Intervention suitability** | **Residual abilities** | | 0.80 |
|  |  | Motor skills | Grasp |  |
|  |  |  | Release and throw objects |  |
|  |  |  | Draw and color with different tools |  |
|  |  |  | Glue and cut |  |
|  |  | Perceptual skills | Handle objects to obtain sensorial information |  |
|  |  |  | Recognize size, shape and color of an object |  |
|  |  | Participation skills | Take part to group discussions and activities when encouraged |  |
|  |  |  | Follow simple instructions |  |
|  |  |  | Pay attention to or bear an activity of interest for 30 minutes |  |
| CR | **Intervention acceptability** | **CRs’ interest to art entertainments** | | n.a. |
|  |  | **Art treatment judgments: CR’s pleasure to participate to an art treatment** | | n.a. |
|  | **Intervention suitability** | **Compliance with art treatment activities** | | 0.68 |
|  |  | Interest in art production activities | Drawing with different tools |  |
|  |  |  | Coloring with different tools |  |
|  |  |  | Gluing |  |
|  |  |  | Cutting with scissors |  |
|  |  | Interest in group activities | Taking part to a discussion |  |
|  |  |  | Taking part to group activities |  |
|  |  | Enjoying art | Listening to music |  |
|  |  |  | Attending to art meetings |  |

**Table 2.** *Ad-hoc* questionnaires: statistics for internal validity. *Abbreviations:* CG, Caregiver; CR, Care Recipient.

Summary statistics of the answers to the two versions (A and B) of the questionnaire are reported in table 3.

| *Questionnaire version* | *Domain* | *Scale* | | *Range* | *Mean* | *SD* |
| --- | --- | --- | --- | --- | --- | --- |
| A - CG | **Intervention acceptability** | CRs’ access to art entertainments* | | 0-12 | 5.94 | 2.46 |
|  |  | Art treatment  judgments | Perceived utility of art treatment for people with cognitive impairment | 1-9 | 6.42 | 2.02 |
|  |  |  | Expected benefit of art treatment for CR | 1-9 | 5.97 | 2.10 |
|  |  |  | Interest of CR in art treatment | 1-9 | 5.11 | 2.37 |
|  | **Intervention suitability** | Residual abilities | Motor Abilities | 0-12 | 8.84 | 2.50 |
|  |  |  | Perceptual Abilities | 0-8 | 6.64 | 1.39 |
|  |  |  | Participation Abilities | 0-12 | 8.47 | 1.90 |
| B - CR | **Intervention acceptability** | CRs’ access to art entertainments* | | 0-12 | 4.43 | 2.34 |
|  |  | Art treatment  judgment | CRs’ pleasure to participate to an art treatment | 1-9 | 5.91 | 2.21 |
|  | **Intervention suitability** | Compliance with art treatment activities | Interest in art production activities | 0-4 | 2.63 | 1.25 |
|  |  |  | Interest in group activities | 0-2 | 1.12 | 0.83 |
|  |  |  | Enjoying art | 0-2 | 1.52 | 0.65 |

**Table 3.** Summary statistics from the *ad-hoc* questionnaires (versions A and B). *Abbreviations:* CG, Caregiver; CR, Care Recipient; SD, Standard Deviation. * Mean scores refer to the number of artistic activities attended by CRs (range = 0-12)

Globally, results showed favorable values for intervention acceptability, with a good CRs’ access to art entertainments and favorable judgments on art-based interventions for these subjects both from CGs’ and CRs’ answers. Correlation analyses between CRs’ and CGs’ opinions on CRs’ access to art entertainments showed a trend of concordance (r = 0.32, p = .06).

As for the suitability of art intervention, CGs’ judged their CRs with sufficient residual abilities to attend an art treatment at a motor and perceptual level as well as for their capacity to participate in a group as depicted in figure 1.


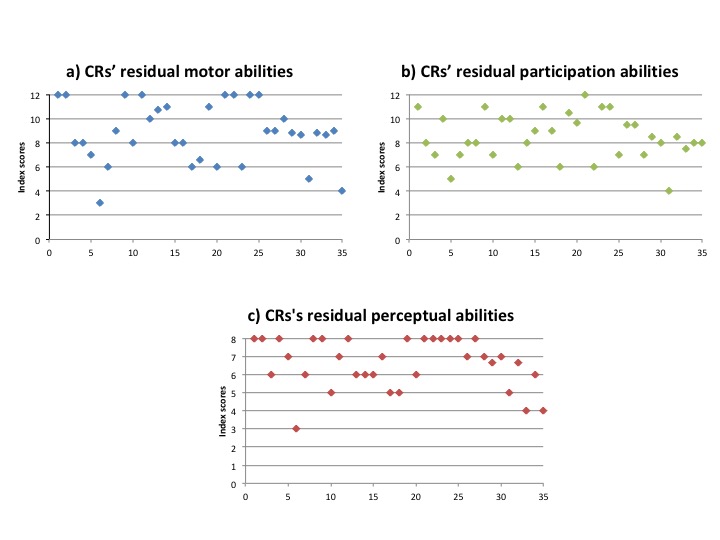


**Figure 1**. Graphical representation of index of a) CRs’ motor (range 0-12), b) participation (range 0-12), and c) perceptual (range 0-8) residual abilities to attend art treatments obtained by CGs’ answers.

CRs also showed interest both in art activities and group participation. Results of the multinomial analyses on the judgments on art treatments are reported in table 4.

| ***Questionnaire version*** | *Indexes* | *Number of answers per scale level*  *N (%)* | | |
| --- | --- | --- | --- | --- |
|  |  | *Low*  *(score range 1-3)* | *Medium*  *(score range 4-6)* | *High*  *(score range 7-9)* |
| **A - CG** | Perceived utility of an art treatment for people with cognitive impairment | 3 (8.82)** | 14 (41.18) | 17 (50)* |
|  | Expected benefit of art treatment for the CR | 3 (9.09)** | 18 (54.54)** | 12 (36.36) |
|  | Interest of CR in an art treatment | 7 (20.59) | 19 (55.88)** | 8 (23.53) |
| **B - CR** | Pleasure in participating to an art treatment | 6 (17.14)* | 16 (45.71) | 13 (37.14) |

**Table 4.** CGs’ and CRs’ judgments on visual art-based interventions for the treatment of mild to moderate stages of AD.*Abbreviations:* CG, Caregiver; CR, Care Recipient; N, number. * observed % > or < 33.3%, *p* < .05; ** observed % > or < 33.3%, *p* < .01.

As for the utility of an art intervention for people with cognitive impairment, the majority of CGs considered an art treatment highly useful, while only a negligible part judged it lowly useful. As for the benefits their CR can obtain from an art intervention and her/his interest to it, the majority of CGs provided medium scores. Concerning the four categories to assess the justification of the interest of CRs’ towards intervention, answers were available for 23 CGs, and the data were analyzed. The justifications of CGs are graphically represented in figure 2. Only a few CGs gave low-rank judgments which, nevertheless, were mostly related to the failure of past rehabilitation group experiences and not to art treatments broadly speaking.


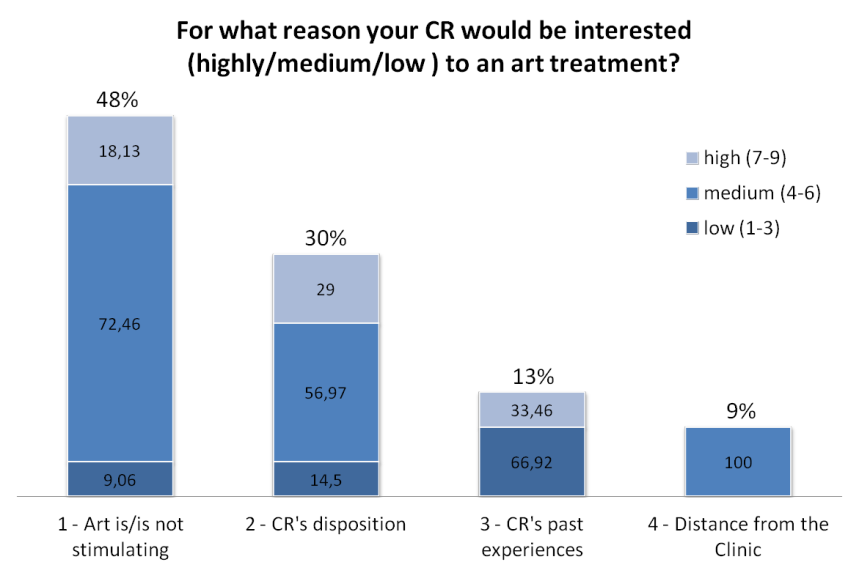


**Figure 2.** For each of the four justification-category about CRs’ interest to an art-based intervention, the percentage of CGs’ answering (N = 23) was reported. The percentage of answers was further subdivided in relation to CRs’ level of interest in art-based interventions judged by CGs (low, medium, high).

# Conclusion

# Concluding, the sample of outpatients and caregivers we interviewed on the acceptability and suitability of an art intervention for people with cognitive impairment gave positive feedback.

# Reference

1. Orsmond, GI, Cohn, ES. The distinctive features of a feasibility study: Objectives and guiding questions. OTJR (Thorofare N J). 2015; 35(3): 169-77.
2. Petersen, RC. Mild cognitive impairment as a diagnostic entity. J Intern Med. 2004; 256 (3): 183-94.
3. Measso G, Cavarzeran F, Zappala G, Lebowitz BD, Crook TH, Pirozzolo FJ, Amaducci LA, Massari D, Grigoletto F. The mini‐mental state examination: normative study of an Italian random sample. Dev Neuropsychol 1993; 9: 77-85.
4. McKhann GM, Knopman DS, Chertkow H, Hyman BT, Jack CR, Kawas CH, Klunk WE, Koroshetz WJ, Manly JJ, Mayeux R, Mohs RC, Morris JC, Rossor MN, Scheltens P, Carrillo MC, Thies B, Weintraub S, Phelps CH. The diagnosis of dementia due to Alzheimer's disease: recommendations from the National Institute on Aging-Alzheimer's Association workgroups on diagnostic guidelines for Alzheimer's disease. Alzheimers Dement. 2011; 7(3): 263-9.
